# Spawning of pacu, *Piaractus mesopotamicus*, at two different times, with a focus on melatonin levels and reproductive performance

**DOI:** 10.1590/1984-3143-AR2025-0024

**Published:** 2026-07-06

**Authors:** Rafael Tomoda Sato, Mariana Roza de Abreu, Laíza Maria de Jesus Silva, Cristiane Fernanda Benevente, Sergio Ricardo Batlouni

**Affiliations:** 1 Centro de Aquicultura da UNESP – CAUNESP, Universidade Estadual Paulista – UNESP, Jaboticabal, SP, Brasil; 2 Centro de Pesquisa, Desenvolvimento e Inovação – CPDI-Norte, Instituto Capixaba de Pesquisa, Assistência Técnica e Extensão Rural – INCAPER, Linhares, ES, Brasil

**Keywords:** circadian rhythm, migratory fish, ovulation rate, spawning

## Abstract

Melatonin (MTN) plays a direct role in fish ovulation, and we hypothesized that aligning ovulation with an endogenous MTN peak levels could improve reproductive performance. To that, in experiment 1, we assessed circadian variation of plasma MTN in pacu (*Piaractus mesopotamicus*) females. In experiment 2, pacu females were divided into two groups: one receiving the hypophysation dose at 7 pm (dark onset) and the other at midnight (five hours after dark). Experiment 1 showed that circulating MTN levels increased at 7 pm and remained stable through the dark phase. In experiment 2, no differences in latency or reproductive performance were observed between groups. However, MTN levels at ovulation increased significantly only in the 7 pm group. Strong positive correlations between MTN levels at ovulation and reproductive parameters, such as fertility, hatching rates, and fecundity, were observed. These results confirm that PGF2α and DHP peaks at ovulation are associated with successful ovulation in pacu, consistent with literature that shows that MTN influences ovulation by acting through the Mtnr1a receptor, triggering arachidonic acid release and prostaglandin synthesis. Our findings indicate that the timing of spawning induction influences MTN levels at ovulation, which are positively correlated with reproductive success. This is the first report linking plasma MTN levels at ovulation to successful reproductive performance in fish induced by hypophysation, highlighting the relevance of spawning timing for pacu reproduction.

## Introduction

Among the native freshwater species farmed in Latin America (LA), "round fishes", named for their characteristic shape, stand out as some of the most widely cultivated. They rank among the largest freshwater fish produced across multiple LA countries. (Mechaly et al., 2024; Batlouni et al., 2024; IBGE, 2025). The pacu (Teleostei, Characiformes, Serrasalmidae) is native to South America, specifically the Paraguay-Paraná river basin (Jégu, 2003). In this species, sexual differentiation is first observed approximately 150 days after hatching, with a mean total length of 10.03 ± 0.12 cm and a mean total biomass of 18.21 ± 1.22 g. (Barbosa et al., 2020). There is no available information on the onset of puberty for this species, but broodstock used in aquaculture typically weighs around 3.0 kg (Schorer et al., 2016; Sato et al., 2020). As a rheophilic species, pacu has a seasonal reproductive cycle, and its reproductive season typically occurs from October to March in Brazil, coinciding with the rainy season and increasing water temperatures (Schorer et al., 2016; Kuradomi et al., 2017).

Our studies on pacu show that ovulation failure is linked to meiosis resumption after hormonal induction, without subsequent ovulation. As a result, follicle-enclosed oocytes remain retained in the ovaries at the GVBD stage (Criscuolo-Urbinati et al., 2012; Kuradomi and Batlouni, 2018; Sato et al., 2023; Abreu et al., 2025). GVBD oocytes clearly indicate final oocyte maturation, marked by meiosis resumption and progression to metaphase II (Nagahama and Yamashita, 2008). These oocytes, characterized by nuclear breakdown and yolk globule fusion, can be observed in the ovaries during unsuccessful spawning (Criscuolo-Urbinati et al., 2012). In this context, we have previously proposed a modified hypophysation protocol using prostaglandin F2α (PGF2α), a follicle rupture inducer, which has been effective in pacu (reviewed in Batlouni et al., 2024). However, PGF2α levels at ovulation did not fully explain spawning failures (Kuradomi and Batlouni, 2018), suggesting the involvement of additional mechanisms that require further study.

A major issue in pacu reproduction is the arbitrary timing of hormonal induction, often based on fish farmers' convenience rather than scientific criteria. For pacu and other migratory species, hormone application usually disregards the circadian rhythms involved in ovulation due to a lack of research. While model species like zebrafish (Carnevali et al., 2011; Yumnamcha et al., 2017) and medaka (Ogiwara and Takahashi, 2016) have defined spawning times, studying the natural spawning time of migratory fish is challenging since they travel long distances and their spawning sites are hard to track (Zaniboni-Filho et al., 2017; Reynalte-Tataje et al., 2024). The limited data available comes from captivity. For tambaqui (*Colossoma macropomum*), Brazil's most farmed native species (IBGE, 2025), morning spawning induction led to higher ovulation rates, while evening induction resulted in 0% spawning (Muniz et al., 2008).

Photoperiod is a key regulator of behavioral and physiological processes, including reproduction (Chattoraj et al., 2005; Falcón et al., 2007, 2010; Alba et al., 2019; Paredes et al., 2019). This variable follows a circadian pattern, alternating light and dark periods over approximately 24 hours, with melatonin (MTN) as its main neurohormonal mediator (Falcón et al., 2007, 2010). MTN modulates vertebrate circadian clocks, with high plasma levels at night and low levels during the day (Falcón et al., 2010; Ogiwara and Takahashi, 2016). In fish, the retina and pineal organ respond to photoperiod both neurally (via the ventral diencephalon) and hormonally (via MTN) (Falcón et al., 2007, 2010). Retinal MTN acts autocrinely and/or paracrinely, while pineal MTN enters the cerebrospinal fluid and blood, binding to receptors in the hypothalamus, pituitary, gonads, liver, and other tissues. MTN appears to directly influence ovulation. In carp oocytes, MTN accelerated 17α-20β-dihydroxy-4-pregnen-3-one (DHP) action when added four hours before DHP *in vitro*, advancing final oocyte maturation (germinal vesicle breakdown — GVBD) (Chattoraj et al., 2005). Similar results were observed in zebrafish follicles, with MTN increasing the GVBD rate when combined with DHP (Carnevali et al., 2011). In *C. catla*, final maturation occurred with MTN alone (Chattoraj et al., 2008). 

Therefore, in the present study we first determined the circadian plasma oscillation pattern of MTN in pacu over 24 hours. As a second objective, we evaluated the reproductive performance and plasma profiles of MTN, PGF_2α_ and DHP of pacu females submitted to hormonal induction in two distinct nocturnal periods routinely used in fish farms: at 7 pm and at midnight. Reproductive performance variables were correlated with the plasma levels of MTN, PGF_2α_ and DHP, allowing us to draw an unprecedented profile for this scenario in tropical freshwater migratory species.

## Methods

This study was conducted in agreement with the precepts of the National Council for the Control of Animal Experimentation (CONCEA) and was approved by the Animal Ethics and Welfare Committee from UNESP, Jaboticabal, SP, Brazil, under permission number 003837/19. The experiments were carried out during the pacu reproductive season, in November 2020 (experiment 1) and December 2021 (experiment 2) ﻿at the UNESP Aquaculture Center (CAUNESP), located in Jaboticabal, São Paulo, Brazil (21º15’17”S and 48º19’20”W). The broodstock was maintained throughout the year in 200 m^2^ earthen ponds (at a density of 0.80 kg/m^2^), supplied with a constant flow of approximately 20 L/min. The fish were fed *ad libitum* twice a day (approximately 1-4% of the biomass) with commercial feed Omnivores Growth – Fri-Acqua, containing 28% crude protein, 3.5% ether extract, 12% ash and 9% of fibrous matter, according to the manufacturer’s information. The fish used were produced at CAUNESP from crossings carried out with the broodstock already existing at the Institution.

Water parameters were determined every two weeks at 7 am. Temperature and conductivity were measured using a HANNA probe, HI-98311; pH was measured using a KASVI probe, K39-0014P and dissolved oxygen with a BERNAUER-424 AQUACULTURE probe, F-1550A. ﻿The mean ± standard deviation of pH, dissolved oxygen concentration, conductivity and water temperature, were, at 6.05 ± 0.72, 6.21 ± 0.42 mg/L, 32.10 ± 15.65 μS/cm and 25.21 ± 3.71 °C, respectively, in experiment 1 and, 6.12 ± 0.31, 5.80 ± 0.55 mg/L, 33.89 ± 11.24 μS/cm and 24.24 ± 3.56 °C respectively, in experiment 2.

### Broodstock selection

In both experiments, females were selected by external traits (swollen abdomen, hemorrhagic urogenital papilla), and males by semen release under slight pressure. Fish were transported in oxygenated water and kept in 750 L tanks with a recirculation system using pond-sourced water. Temperatures were stable (26.78 ± 0.22°C in experiment 1; 27.04 ± 0.25°C in experiment 2). Each fish was considered an experimental unit.

#### Hormonal induction protocol

Hormonal induction was divided into two intramuscular injections with an interval of 24 hours between them. Prior to hormonal induction, females were anesthetized ﻿with a 100 mg/L of benzocaine solution (Sigma-Aldrich, Saint Louis, USA). Females received two doses of crude carp pituitary extract (CPE) (0.6 and 5.4 mg/kg) suspended in 0.25 mL/kg of 0.9% saline solution. The saline group only received 0.9% saline solution in both injections at the same volume (i.e., 0.25 mL/kg), while the control group did not receive any injection during the experimental period. In experiment 2, exogenous PGF_2α_ induction was performed via intramuscular injection of 2 mL of Ciosin^®^ per fish (containing 0.25 mg/mL of cloprostenol, MSD Animal Health) at the time of resolving doses. In experiment 2, males received 3 mg/kg of CPE at the time of the resolving dose for females.

### Experiment 1

Experiment 1 aimed to determine the circadian pattern of plasma MTN oscillation over 24 hours. The effects of hypophysation on MTN levels remain unknown; however, GnRH treatment has been shown to increase MTN levels at night in *Dicentrarchus labrax* (Servili et al., 2010), suggesting that hormonal induction may modulate MTN circadian dynamics. As pacu spawning relies on hormonal induction, mainly via hypophysation, we evaluated the potential effect of CPE on MTN circadian rhythm. The conventional CPE protocol (without PGF2α) was used, as it remains the standard method among farmers (Batlouni et al., 2024). Given MTN role in prostaglandin synthesis (Ogiwara and Takahashi, 2016), exogenous PGF2α was excluded to prevent interference.

**Figure 1:**
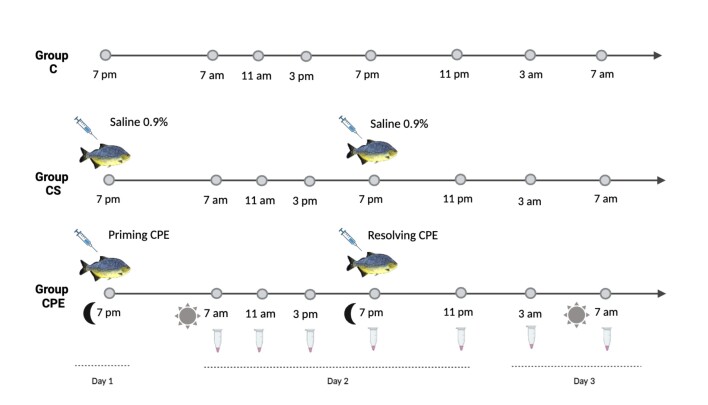
Experiment 1. *Piaractus mesopotamicus* females were distributed into three groups: C (control – fish were not handled); CS (saline control – fish received 0.9% saline solution in both injections); and CPE (carp pituitary extract) (two doses classical hypophysation) (n=6 females per group). Seven samplings were performed every four hours between 7 am on day two and 7 am on day three (represented by microtubes). The moon represents the beginning of the night, and the sun represents the beginning of the day. Created in BioRender.

Eighteen adult females with a body mass (mean ± SEM) of 3,412.50 ± 192.89 g were distributed in a completely randomized design into three groups (n = 6 females per group): control group I, fish were not handled; saline control (SC), fish received 0.9% saline solution in both injections; and classic hypophysation with CPE (Figure 1). During Experiment 1, sunrise (the beginning of the day) occurred at 5:14 AM, and sunset (the beginning of the night) at 6:29 PM, resulting in a total daylength of 13 hours and 11 minutes and a dark period of 10 hours and 49 minutes. Due to the continuous management of the females throughout hypophysation, we did not conduct analyses of reproductive performance.

#### MTN plasma levels quantification

Blood was collected every four hours from 7 am on day two to 7 am on day three (seven collections per fish, Figure 1). Fish were anesthetized with 100 mg/L benzocaine (Sigma-Aldrich, USA), and blood was drawn from the caudal vein using heparinized syringes. Plasma was separated by centrifugation (1000×g, 15 min, 4 °C) and stored at -80 °C. MTN concentrations were measured by ELISA (IBL International, Germany) following the manufacturer’s instructions. Absorbance was read at 405 nm using an Epoch2 plate reader (BioTek, USA), with all samples analyzed in duplicate. Intra- and inter-assay variability was maintained within 20%.

### Statistical analysis

﻿Statistical tests and graphs were performed using STATISTICA software (StatSoft, Inc., Tulsa, OK, USA) and GraphPad Prism 9 (GraphPad Software Inc.), respectively. Normality and homogeneity of the variances were tested using Shapiro-Wilk and Levene’s test, respectively. MTN plasma levels showed normal distribution and homoscedasticity being compared by repeated measures ANOVA test followed by a multiple comparisons test (Fisher LSD). Significant differences were accepted at a p-value <0.05. Data were expressed as mean ± standard error of the mean (SEM).

### Experiment 2

Unlike the experiment 1, the experiment 2 took a practical approach, focusing on reproductive performance with minimal fish handling, as hypophysation did not affect MTN levels (experiment 1, present study). Building on advancements in pacu-induced ovulation (Criscuolo-Urbinati et al., 2012; Kuradomi and Batlouni, 2018; Sato et al., 2020), we revisited the hypophysation + PGF2α protocol, aiming to refine the method and to explore the effect of induction time — a previously unevaluated variable.

#### Experimental design

**Figure 2:**
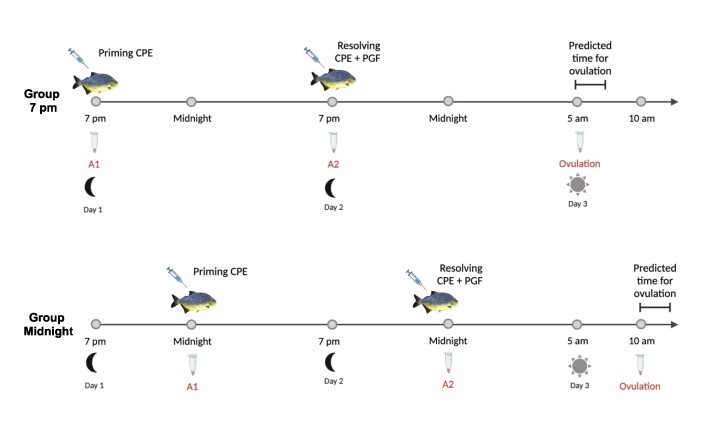
Experiment 2. Details of experimental design of different times of hypophysation associated with prostaglandin F2α (PGF2α) in *Piaractus mesopotamicus* females distributed into two groups: induction at 7 pm (group 7 pm) and induction at midnight (group midnight). The moon represents the beginning of the night, and the sun represents the beginning of the day. CPE: Carp pituitary extract. PGF: prostaglandin F2α. Create in BioRender

Eight adult females with a body mass (mean ± SEM) of 3,176.25 ± 292.17 g were distributed in a completely randomized design into two groups (n = 4 females per group): the 7 pm group and the midnight group. Both groups underwent classic hypophysation associated with exogenous PGF_2α_ (Criscuolo-Urbinati et al., 2012). The priming and resolving doses were applied at 7 pm and at midnight, respectively, in the 7 pm and midnight group (with a one-day interval between injections) (Figure 2). The experiment lasted three days (Figure 2), with sunrise occurring at 5:19 AM and sunset at 6:55 PM. Consequently, the total day length was 13 hours and 36 minutes, and the dark period lasted 10 hours and 24 minutes. The expected ovulation time was estimated to occur between 10 and 12 hours after the resolving dose at 27°C (Criscuolo-Urbinati et al., 2012).

#### Reproductive performance

When females exhibited typical signs of imminent ovulation, such as restlessness and abdominal muscle spasms (Ceccarelli et al., 2000) or the first oocytes released at the bottom of the tanks (Batlouni et al., 2024), abdominal massage was used to extrude the eggs (between 260 and 340 accumulated thermal units (ATU)). ATU were calculated by summing the water temperature (°C) over time (hours) from the resolving dose to ovulation, serving as a predictor for ovulation timing (Figure 2). Oocytes were stripped into a dry container, and both oocyte mass and spawning time were recorded for each female. Females with fewer than 35,000 oocytes per kg of body weight were classified as having poor-quality spawning, showing partial ovulation failure and releasing oocytes in "clumps" mixed with blood, which reduced fertility and hatching rates (Sato et al., 2020). Fertilization was performed using pooled semen from three males (0.5 mL per 50 g of oocytes). Sperm concentration in pacu ranges from 4.7 to 6.9 x 10^10^ cells/mL (Kuradomi et al., 2016), and the average number of oocytes per gram of spawn is approximately 1,200 (Ceccarelli et al., 2000). The fertilized oocytes were hydrated by adding water after mixing with gametes. Then, 10 mL of eggs from each female were transferred to 6.5 L acrylic incubators at an average temperature of 27.2 ± 0.3ºC, using the same recirculated water system for the breeders. Data were collected on ovulation rate, latency (hours), ATU, relative fecundity, and estimated fertility (blastopore closure, ~8 h post fertilization) and hatching rates (tail moving and fully unfolded, ~17 h post fertilization). For fertility and hatching rates, approximately 100 eggs from each incubator (triplicates) were classified as viable or non-viable embryos. The following parameters were recorded:

- **Ovulation rate (%)** = (Number of ovulated females / Total number of females) × 100
- **Latency period** = Time interval between the resolving dose and ovulation, determined individually for each female.
- **ATU** = Mean water temperature (°C) × Latency period (h). As all fish were maintained in a recirculating water system at a constant temperature, the average value of 27.2°C was used for the calculation.
- **Relative fecundity** = Number of released oocytes / Female body mass (kg).
- **Fertility rate (%)** = (Number of viable embryos / Total number of eggs) × 100.
- **Hatching rate (%)** = (Number of viable embryos at 17 h post-fertilization / Total number of eggs) × 100.

#### Plasma levels quantification of DHP, PGF_2α_ and MTN

Blood collections were performed at three times: at the time of the first dose (A1); at the time of the resolving dose (A2); and at the time of ovulation (Figure 2). The blood collection procedure and its storage were performed as described in experiment 1. For PGF_2α_ dosage, 10 μM indomethacin was added to the blood immediately after collection to inhibit prostaglandin synthesis. ﻿The plasma levels quantification was carried out by ELISA using commercial kits for DHP and PGF_2α_ (Cayman Chemical Company, Ann Arbor, MI, USA) and MTN (IBL International, Hamburg, Germany) following the manufacturer’s instructions. The readings of DHP, PGF_2α_ and MTN plates were performed at an absorbance of 412, 405 and 405 nm, respectively, using an Epoch2 plate reader (BioteK Instruments, Inc., Highland Park, Winooski, USA), and all samples were read in duplicate. To validate the analysis, intra- and inter-assay variability were considered up to 20%.

#### Statistical analysis

The statistical tests were performed with the same software used in experiment 1, as well as assumptions such as normality and homoscedasticity. The mean reproductive performance of spawned females (including females with poor-quality spawning) showed normal distribution and homoscedasticity and were compared by the t-test. Plasma levels were submitted to correlations with reproductive performance parameters (regardless of the group) using Spearman's nonparametric rank correlation test. In the correlation event, negligible correlation is considered with ρ (rô) values from 0.0 to 0.10, weak from 0.10 to 0.39, moderate from 0.40 to 0.69, strong from 0.70 to 0.89 and very strong from 0.90 to 1.0, according to a conventional method of interpreting correlation coefficients. ﻿Significant differences were accepted with a P value <0.05. Data were expressed as mean ± SEM and correlation in ρ value.

## Results

### Experiment 1

#### MTN plasma levels

**Figure 3:**
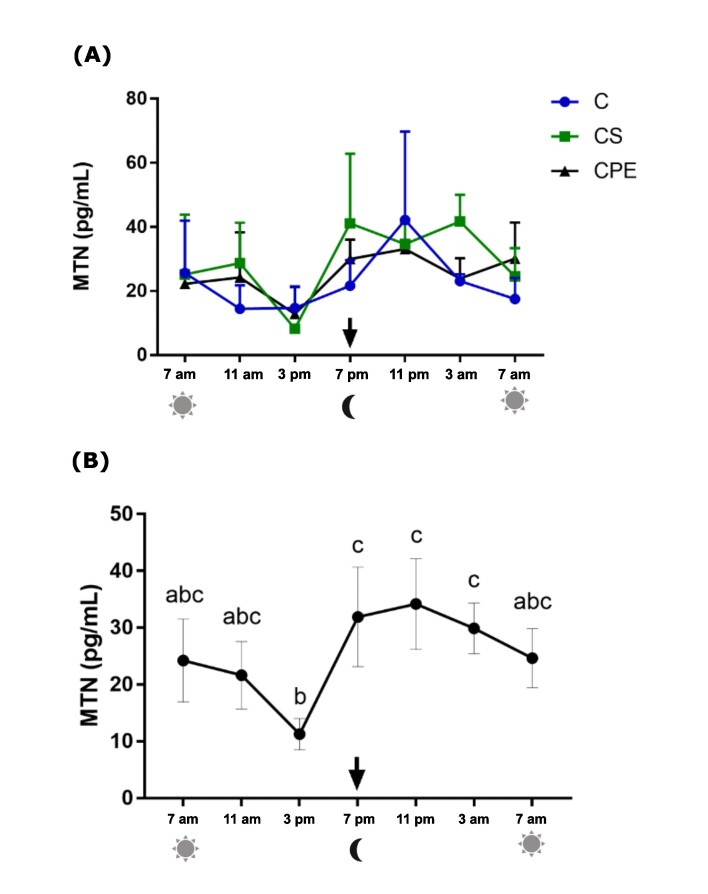
Experiment 1. (A) Circadian plasma profile of melatonin (MTN) in *Piaractus mesopotamicus* females from three groups: C (control, no handling), CS (saline control, 0.9% saline in both injections), and CPE (carp pituitary extract, two doses). Sampling occurred from 7 am on day one to 7 am on day two (seven collections, n=6 per group). Data are mean ± SEM; no significant differences among groups at any time point (p>0.05). The arrow marks the resolving dose. (B) Circadian plasma profile of MTN in P. mesopotamicus females (n=18) over the same period. Data are mean ± SEM; different letters indicate significant differences over time (p<0.05). The arrow marks the resolving dose. Created d in BioRender.

We first observed that the circadian rhythm of MTN in plasma was similar between groups. Statistical differences were not found comparing data from each group at the same collection points (p>0.05) (Figure 3A). Therefore, data from the three groups were pooled, including all fish (n = 18 adult females from the C, SC and H groups), to evaluate the circadian rhythm of plasma MTN (Figure 3B) with a larger sample size. Since no statistical differences were observed among treatments (Figure 3A), we excluded the treatment factor and evaluated the circadian rhythm of MTN over 24h considering all females regardless of treatment. Plasma MTN levels remained similar from early to late morning (7 am – 11 am) (p > 0.05; 22.46 ± 5.70 pg/mL), when these levels begin to decrease, reaching their minimum concentration at 3 pm, and then increasing again in the early evening (7 pm) (p < 0.05; 31.91 ± 6,05 pg/mL), remaining high throughout the dark period (Figure 3B).

### Experiment 2

#### Plasma levels of PGF_2α_, DHP and MTN

**Figure 4:**
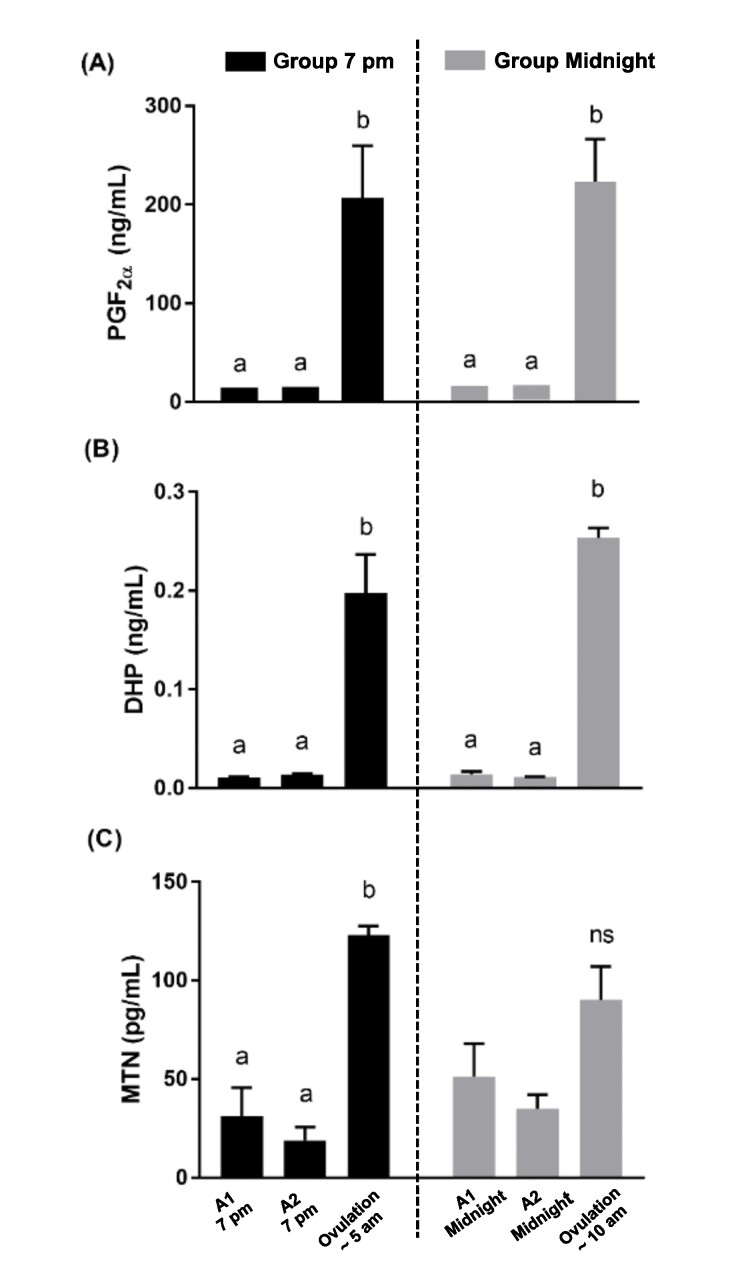
Experiment 2. Plasma levels of (A) prostaglandin F2α (PGF2α), (B) 17α-20β-dihydroxy-4-pregnen-3-one (DHP) and (C) melatonin (MTN) in *Piaractus mesopotamicus* females at the time of the priming dose (A1); the resolving dose (A2); and ovulation (n=4). 7pm: hypophysation at 7 pm; Data expressed as mean ± SEM. Different letters indicate significant differences among collections in the same group during the time (p<0.05). There were no significant differences among groups in any sampling (p>0.05). ns: non significative

There were no differences in the plasma levels of PGF_2α_, DHP and MTN between the groups 7 pm and midnight regardless of the time of collection (p > 0.05) (Figure 4). At ovulation, PGF_2α_ levels were higher than those observed at collections A1 (≈1,300-fold) and A2 (≈1,200-fold) (p<0.05) (Figure 4A). Similarly, DHP levels were higher at ovulation compared to those measured at collections A1 (≈17-fold) and A2 (≈ 14-fold) (p<0.05) (Figure 4B). Regarding MTN, females in the midnight group showed similar levels across all sampling points, whereas females in the 7 pm group exhibited higher levels at ovulation compared to collections A1 (≈4-fold) and A2 (≈6-fold) (p<0.05) (Figure 4C).

#### Reproductive performance

Reproductive performance parameters were similar between the 7 pm and midnight groups (Table 1). The 7 pm group, which stayed longer between resolving dose and ovulation in the dark (from 7 pm to 5:19 am – about 10 hours) showed 75% of ovulation rate, while the midnight group, which stayed approximately 5 hours in the dark after resolving dose (from midnight to 5:19 am), showed 50% of ovulation rate. Females in the 7 pm group did not show poor-quality spawning (i.e., <35,000 oocyte/kg). Relative fecundity, ATU, fertility and hatching rates, and the number of hatched larvae/kg fish were similar between groups (p>0.05) (Table 1). In Table 2, it is possible to observe an absence of an obvious relationship between most of the variables tested with the treatments used (7 pm and midnight), except for the MTN values at the time of spawning. Only two females spawned in the midnight group and the female that presented poor quality spawning was also from this group. In these females, low fertility rates, hatching rate and hatched larvae/kg of fish are also observed. Female 6 was also the one with the lowest individual MTN value.

**Table 1:** **Experiment 2.** Reproductive performance of *Piaractus mesopotamicus* submitted to hormonal induction at two different times.

**Groups**	**Ovulation rate**	Poor-quality spawning*	**ATU**	**Relative fecundity (oocytes/kg of fish)**	**Fertility rate (%)**	**Hatching rate (%)**	**N° of hatched larvae/kg fish**
7 pm	3/4 (75%)	0/3 (0%)	309.7 ± 12.8	114,120 ± 17,498.7	80.0 ± 15.3	71.3 ± 18.4	86,236 ± 28,414
Midnight	2/4 (50%)	1/2 (50%)	305.0 ± 5.8	77,820 ± 44,820	58.5 ± 33.4	49.0 ± 36.2	54,346 ± 50,126

7 pm: application of resolving dose of hypophysation + prostaglandin F_2α_ (PGF_2α_) at 7 pm; midnight: application of resolving dose of hypophysation + PGF_2α_ at midnight. ATU: Accumulated thermal units. Data expressed by mean ± SEM. *occurrence of a female with “poor-quality spawning” (fecundity < 35,000 oocyte/kg fish). ATU: accumulated thermal units. ATU = mean of water temperature (°C) × latency period (h). Latency period = Interval between the time of resolving dose and ovulation determined individually for each female. Since all fish were maintained in a recirculated water system with same temperature, we used the average value of 27,2^o^C for calculating ATU.

**Table 2:** **Experiment 2.** Individual analysis of reproductive performance and plasma hormone levels of *Piaractus mesopotamicus* females at the time of ovulation and submitted to hormonal induction at two different times.

**Female**	**Groups**	**Latency period**	**ATU**	**Relative fecundity**	**Fertility rate (%)**	**Hatching rate (%)**	**N° of hatched larvae/kg fish**	**PGF_2α_ (ng/mL)**	**DHP (ng/mL)**	**MTN (pg/mL)**
1	7 pm	12h8min	330.0	83,400	49.6	35.5	29.591	167.24	0.16	119.29
2	7 pm	11h29min	312.2	144,000	92.2	82.3	118.512	119.16	0.16	123.74
3	7 pm		-	-	-	-	-	177.37	0.31	113.57
4	7 pm	10h30min	286.0	114,960	98.2	96.2	110.605	362.26	0.16	135.17
5	Midnight		-	-	-	-	-	351.66	0.26	91.47
6*	Midnight	11h25min	310.8	33,000*	25.1	12.8	4.219	170.23	0.25	42.51
7	Midnight		-	-	-	-	-	182.34	0.28	116.22
8	Midnight	11h	299.2	122,640	91.9	85.2	104.472	188.96	0.22	110.76

Females underwent hypophysation with two doses (0.6 mg/kg and 5.4 mg/kg) 24 hours apart, with PGF2α added to the resolving dose. ATU: accumulated thermal units, calculated as water temperature (°C) × latency period (h). Latency period: time from the resolving dose to ovulation, determined individually. All fish were kept in a recirculated water system at 27.2°C. Data are mean ± SEM. *Poor-quality spawning: fecundity < 35,000 oocytes/kg. Resolving dose + PGF2α applied at 7 pm or midnight.

#### Correlation

Correlations between variables were analyzed regardless of group. Positive correlations from strong to moderate were observed between plasma levels of MTN at the time of ovulation and either fertility rate or hatching rate or number of larvae per kg of fish and relative fecundity (Table 3). Furthermore, negative and strong correlations were found between PGF_2α_ x ATU, ATU x hatching rate (Table 3).

**Table 3:** **Experiment 2.** Spearman's correlation.

**Correlation**	**ρ**	**Rate**
MTN x fertility rate	0.90	Strong
MTN x hatching rate	0.70	Strong
MTN x nº larvae/kg fish	0.80	Strong
MTN x fecundity	0.60	Moderate
PGF_2α_ x ATU	-0.90	Strong
ATU x hatching rate	-0.70	Strong

MTN: melatonin; Fertility and hatching rate: number of viable embryos/total numbers of eggs; Fecundity: number of oocytes released/kg fish; PGF_2α_: Prostaglandin _2α_; ATU: Accumulated thermal units. Rate: following (Schober et al., 2018). ATU: accumulated thermal units. ATU = mean of water temperature (°C) × latency period (h). Latency period = Interval between the time of resolving dose (in hours) and ovulation determined individually for each female. Since all fish were maintained in a recirculated water system with same temperature, we used the average value of 27,2^o^C for calculating ATU. Data expressed by mean ± SEM.

## Discussion

MTN, a neurohormone synthesized by the pineal gland, follows a circadian rhythm and regulates reproduction by influencing GnRH release in the hypothalamus and binding to ovarian granulosa cells (Ogiwara and Takahashi, 2016; Hasan et al., 2023; Takahashi and Ogiwara, 2021). Our study characterized the circadian rhythm of pacu MTN plasma levels. A peak in plasma MTN was observed at the onset of the dark phase (between 3 PM and 7 PM), with levels remaining significantly elevated until 3 AM, consistent with a Type C plasma MTN pattern (Falcón et al., 2010). Moreover, the circadian rhythm of plasma MTN in pacu was not influenced by CPE treatment. Although the role of MTN in teleost reproduction has been demonstrated both *in vivo* (Carnevali et al., 2011; Falcón et al., 2010; Moniruzzaman et al., 2016; Hasan et al., 2023) and *in vitro* (Chattoraj et al., 2005; Ogiwara and Takahashi, 2016; Takahashi and Ogiwara, 2021), its precise mechanism in regulating final maturation and ovulation remains to be fully understood.

No significant differences in MTN plasma levels were observed among pacu females treated with hypophysation (experiment 1), with similar concentrations across control and carp pituitary extract-treated groups, sampled every 4 hours over 24 hours. On the other hand, Servili et al. (2010) showed that GnRH increased MTN levels at night, both *in vivo* and in *vitro*, in *Dicentrarchus labrax*, linked to GnRH-2 fibers in the pineal gland expressing the dlGnRHR-II-2b receptor. GnRH-2 and GnRHa both stimulated pineal MTN release. More recently, Moniruzzaman et al. (2016) found that two doses of GnRH did not raise MTN levels in ovarian tissue at ovulation, though exogenous MTN injection did. Overall, the effects of hormonal treatments on MTN levels in fish remain limited and poorly understood, being necessary to be investigated (Barades et al., 2025; Byun et al., 2025).

MTN levels in pacu decreased during the day (light phase) and increased at the onset of the night, consistent with the conserved circadian rhythm of circulating MTN observed in other vertebrates (Maitra et al., 2013; Takahashi and Ogiwara, 2021). This plasma MTN profile aligns with the "Type C" pattern described by (Falcón et al., 2010), characterized by a prolonged MTN peak that persists throughout most of the dark phase. The basal circulating MTN level (mean of 11.3 pg/mL) and higher values (ranging from 29.9 to 34.2 pg/mL) during the light and dark periods were similar to those reported in other tropical species, such as Nile tilapia and African catfish (Martinez-Chavez et al., 2008).

In the second experiment, we evaluated reproductive performance under two different CPE injection schedules (7 pm and midnight). Although reproductive performance was similar between the 7 pm and midnight groups, a novel finding was the increase in MTN levels at the time of ovulation only in the 7 pm treatment. This observation suggests several possibilities: a) The increase in MTN levels at 6 am (early light phase – time of ovulation) in the 7 pm group, compared to A1 and A2 samplings, may be related to ovulation and/or follicle rupture, as previously reported (Sato et al., 2023); b) The increase in MTN at the time of ovulation in the 7 pm treatment may be associated with ovulation occurring during the dark period (~ 5 am). Further studies are needed to clarify the cause of this increase in MTN during spawning. However, although spawning also occurred in the midnight group, no increase in MTN was observed at the time of spawning in that group (females that spawned in the late morning). This provides additional evidence for the influence of the light/dark cycle, which warrants further investigation.

Additionally, Table 2 shows greater variability in reproductive performance for the midnight group, with more than double the standard error compared to the 7 pm group, suggesting less stable reproductive outcomes. All MTN values in the midnight group were lower than in the 7 pm group, except for one female. At ovulation, MTN ranged from 42.51 to 116.22 pg/mL (midnight) and 113.74 to 135.17 pg/mL (7 pm), contributing to similar MTN levels across samplings in the midnight group (A1 and A2) despite visibly high values (Figure 4).

Considering the absolute number of larvae, a key variable for fish farmers, the 7 pm group achieved nearly a threefold increase in larval production compared to the midnight group (737,567 vs. 250,762, respectively), with the same number of induced females (ovulation rates of 3/4 and 2/4, respectively). Several observations can be made from this result. While no differences were found between the 7 pm and midnight groups in terms of reproductive performance variables, it is important to note that the 7 pm treatment exposed the fish to a longer dark period between the second hormonal dose and ovulation (from 7 pm to 5 am, resulting in a 10-hour dark period). In contrast, the midnight group had approximately 6 hours of darkness (4 hours less than the 7 pm group). This interpretation is supported by recent evidence from other fish species, indicating that elevated melatonin availability is associated with improved reproductive outcomes, including enhanced cellular regulation in oocytes and maintenance of fertilization capacity under conditions of reproductive stress (Jabbari et al., 2025).

Although these differences did not affect the reproductive performance of pacu in this study, a significant effect was observed in tambaqui (*Colossoma macropomum*). Muniz et al. (2008) reported a significant advantage in spawning rate for tambaqui induced to ovulate between 6-8 am, compared to those induced between 5-7 pm. Given that pacu are often induced to ovulate during the day in many fish farms, it would be valuable to conduct a follow-up study inducing pacu females to spawn both in the morning and at night, to determine whether these light/dark period differences could improve reproductive performance, as seen in tambaqui, particularly regarding spawning success and reduced anomalies. It is also important to note that many studies on spawning induction do not specify the timing of hormonal treatments, which could be a key factor in optimizing reproductive outcomes.

Successful ovulation in pacu has been previously shown to correlate with concomitant peaks in PGF2α and DHP at the time of ovulation (Kuradomi and Batlouni, 2018; Sato et al., 2020, 2023). In this study, we reaffirmed these findings while also presenting new evidence regarding the role of MTN. Specifically, we observed increased levels of MTN, DHP, and PGF at the time of ovulation. Moreover, MTN levels at ovulation were positively correlated with fertility rate, hatching rate, and fecundity, while ATU values showed a strong negative correlation with these same reproductive parameters. This corroborates that higher ATU values are associated with reduced reproductive success (Kuradomi and Batlouni, 2018; Sato et al., 2020). These findings were further supported by the negative correlation between PGF2α levels and ATU, and between ATU and hatching rate observed in the present study. Therefore, a shorter latency period (or ATU, considering temperature) remains one of the primary indicators of successful reproductive performance in this species, although the underlying reasons for variability among females remain unknown. In essence, a shorter interval between spawning and ovulation correlates with higher PGF2α levels and improved hatching rates.

The role of MTN in ovulation, though not fully understood, is supported by studies in other fish species. In carps, MTN injection reduced latency and accelerated GVBD (Moniruzzaman et al., 2016; Sudrajat et al., 2025). In medaka, the MTN inhibitor luzindole suppressed ovulation when applied 12 hours before ovulation (Ogiwara and Takahashi, 2016). In zebrafish, MTN increased the GVBD rate (Carnevali et al., 2011). In medaka, MTN binding to Mtnr1a likely induces Pla2g4a expression, releasing arachidonic acid, which is converted into PGE2. MTN modulates the hypothalamic-pituitary-gonadal axis, signaling from kisspeptin (Carnevali et al., 2011; Maitra et al., 2013), contributing to final oocyte maturation via DHP synthesis (Chattoraj et al., 2008; Carnevali et al., 2011) and ovulation through prostaglandin pathways and moesin A phosphorylation (Takahashi et al., 2013, 2018; Hagiwara et al., 2014). These mechanisms highlight both the classical role of plasmatic MTN on the HPG axis and its paracrine action in the ovary. Since this information is unavailable for pacu or other South American aquaculture species, further *in vitro* and *in vivo* studies on MTN direct role in ovulation are essential for Neotropical rheophilic species.

## Conclusion

In conclusion, serum melatonin (MTN) levels in pacu exhibit a circadian rhythm, with higher concentrations observed during the night compared to daytime. Hypophysation did not alter serum MTN levels compared to controls. Both 7 pm and midnight hormonal induction treatments resulted in similar reproductive performance; however, MTN levels increased only during the 7 pm treatment, at the time of spawning, with ovulation occurring at 5 am. Regardless of the group, MTN levels at ovulation were positively correlated with all reproductive performance variables. Although the role of MTN in reproduction is not yet fully understood in pacu and other migratory species, the results of this study indicate its potential applicability in improving reproductive performance.

## Data Availability

Research data is available in the body of the article.
